# Breast cancer-related fatigue: Mechanisms, biomarkers, risk factors, and intervention strategies

**DOI:** 10.1016/j.breast.2026.104905

**Published:** 2026-08-18

**Authors:** Chaofan Zhao, Qi Qiu, Yuanyuan Wang, Yidi Jiang, Kunshuang Shen, Le Yang, Ye Sun, Zhengcan Zhou, Mingqian Jia, Haibin Liu, Hui Sun, Ning Zhang

**Affiliations:** a State Key Laboratory of Integration and Innovation of Classic Formula and Modern Chinese Medicine, National Chinmedomics Research Center, National TCM Key Laboratory of Serum Pharmacochemistry, Metabolomics Laboratory, Department of Pharmaceutical Analysis, Heilongjiang University of Chinese Medicine, Heping Road 24, Harbin, 150040, China; b State Key Laboratory of Dampness Syndrome, The Second Affiliated Hospital Guangzhou University of Chinese Medicine, Dade Road 111, Guangzhou, China; c Shandong Key Laboratory of Gelatin-Based Drug Research and Development, National Engineering Technology Research Center for Gelatin-based TCM, Shandong Provincial Engineering Research Center of Gelatin-Based Traditional Chinese Medicine for Technology Innovation and Application, Dong'e Ejiao Co., Ltd., Liaocheng, 252200, China

**Keywords:** Breast cancer-related fatigue, Pathogenesis, Risk factors, Interventions, Biomarkers, HPA axis

## Abstract

Breast cancer-related fatigue (BCRF) is a prevalent and debilitating condition that affects patients during and after treatment. It is characterized by persistent, distressing physical and psychological exhaustion that is disproportionate to recent activity and unrelieved by rest. This review synthesizes current evidence on the pathogenesis, biomarkers, risk factors, and intervention strategies for BCRF. Mechanistically, BCRF arises from a complex interplay between inflammation, hypothalamic–pituitary–adrenal (HPA) axis dysregulation, mitochondrial dysfunction, neuromuscular impairment, and psychosocial influences. Multiple biomarkers, including cytokines, cortisol, leptin, and mitochondrial DNA, offer diagnostic and prognostic potential. Risk factors span genetic predisposition, psychological distress, lifestyle behaviors, social stressors, and treatment-related variables. A range of interventions—including acupuncture, exercise, psychotherapy, pharmacotherapy, nutritional support, and microbiota modulation—have demonstrated varying degrees of efficacy. We highlight the need for mechanism-specific, personalized interventions and propose directions for future research and clinical practice.

## Highlights

• Comprehensive integration of BCRF mechanisms from cellular to psychosocial levels.
• Identification of diverse biomarkers for early detection and targeted therapy.
• Multimodal interventions matched to underlying pathophysiology.
• Roadmap for future personalized, mechanism-based clinical trials.

## Graphical abstract

**Figure:**
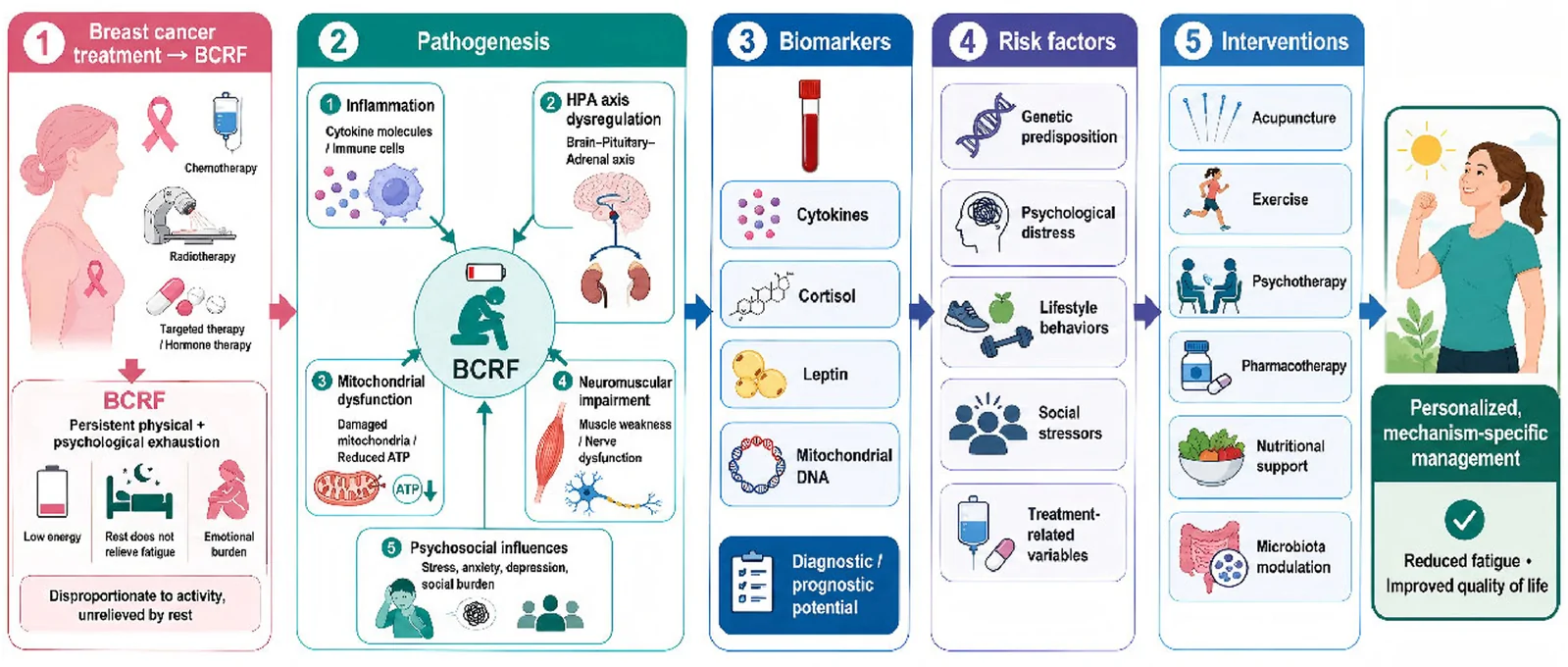

## 1 Introduction

Cancer-related fatigue (CRF) is defined by clinical guidelines as a distressing, persistent, and subjective sense of physical, emotional, and/or cognitive tiredness related to cancer or its treatment. This fatigue is disproportionate to recent activity and interferes with normal functioning. CRF is more severe, distressing, and less alleviated by rest than the fatigue experienced by healthy individuals [1]. Studies report a 49% prevalence of fatigue among cancer patients, with higher rates observed during active treatment [[1], [2], [3]]. For cancer survivors, fatigue persists as a debilitating symptom for months to years after treatment [[4], [5], [6], [7], [8], [9], [10], [11], [12], [13], [14]]. Persistent CRF significantly impairs quality of life [[14], [15], [16], [17]] and delays return to work [18].

The incidence of CRF varies based on cancer type [12], gender [19], stage [5], treatment status [20], and time since treatment [5]. Evidence suggests that breast cancer survivors (BCS) experience a higher prevalence of CRF than survivors of other cancer types [12,21,22]. Approximately one-third of long-term BCS report chronic fatigue, with one-fourth experiencing persistent fatigue lasting more than 10 years [23]. Fatigue intensifies during cytotoxic therapies (chemotherapy, radiation, surgery) [5,[23], [24], [25], [26], [27], [28]] and can persist for years after treatment [26,27]. CRF negatively impacts work capacity, social relationships, emotional well-being, daily activities, and overall quality of life during and after treatment [26]. Patients often report CRF as more severe, persistent, and debilitating than "normal" fatigue, which remains unrelieved by adequate rest [29].

Breast cancer-related fatigue (BCRF) is highly prevalent and exerts significant negative impacts. Due to its multifactorial etiology and complex underlying mechanisms, current clinical management strategies—though diverse—lack systematic integration, which presents challenges for diagnosis and treatment. Therefore, this study aims to: 1) summarize existing evidence on the biological mechanisms underlying BCRF; 2) investigate its potential biomarkers and risk factors; and 3) review effective interventions. These objectives are pursued with the goal of providing evidence-based management strategies for clinical practice.

## 2 The pathogenesis of BCRF

**Fig. 1:**
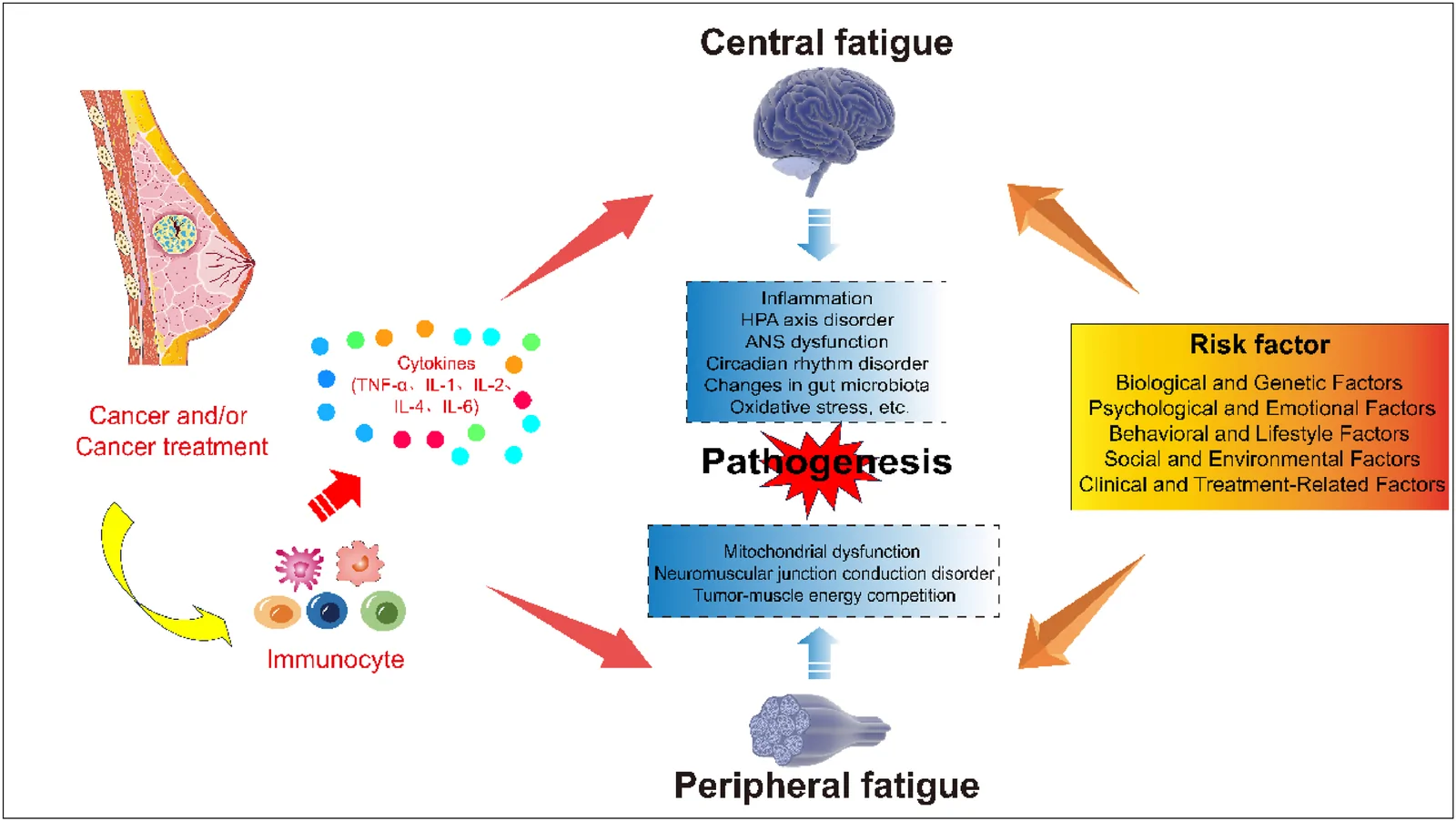
Pathophysiological Mechanisms of Cancer-Related Fatigue in Breast Cancer. HPA: hypothalamic–pituitary–adrenal, ANS: autonomic nervous system, TNF-α: tumor necrosis factor-α, IL-1: interleukin-1, IL-2: interleukin-2, IL-4: interleukin-4, IL-6: interleukins-6.(Note: Some image elements were adapted from https://bioicons.com/.).

BCRF manifests through two primary mechanisms: central and peripheral fatigue. Central fatigue is primarily mediated by inflammation, HPA axis dysfunction, autonomic nervous system (ANS) dysregulation, circadian rhythm disruption, and alterations in the gut microbiota. In contrast, peripheral fatigue is mainly attributed to mitochondrial dysfunction. Other contributing factors include the endocannabinoid system (ECS), dietary influences, oxidative stress, lipid accumulation, and functional changes in specific brain regions. Among these, persistent inflammation during disease progression and treatment plays a central role in mediating the aforementioned pathophysiological processes(Fig. 1).

### 2.1 Central fatigue mechanism

#### 2.1.1 Inflammation

Sustained inflammation plays a pivotal role in the development of CRF among breast cancer patients. Several inflammatory mediators—including eotaxin, monocyte chemoattractant protein-1 (MCP-1), vitamin D binding protein (VDBP), granulocyte-macrophage colony-stimulating factor (GM-CSF), interleukins (IL-1α, IL-2, IL-4, IL-6, IL-8, IL-10, IL-12, IL-13, IL-15), interferon-γ (IFN-γ), and tumor necrosis factor-β (TNF-β)—are significantly correlated with BCRF [[30], [31], [32], [33]]. Biomarkers such as C-reactive protein (CRP), IL-6, and interferons may contribute to central fatigue by inducing anemia and cachexia [[34], [35], [36], [37], [38]]. In addition, transcriptional upregulation of inflammation-related genes, particularly those regulated by the nuclear factor kappa B (NF-κB) pathway, can promote fatigue-related signaling [39]. Recent studies also indicate that IL-15 plays a critical role in breast cancer-induced peripheral muscle fatigue. Preclinical models show that mice bearing advanced tumors exhibit lower IL-15 expression and more severe muscle fatigue than those with early-stage tumors or tumor-free controls [40]. Overexpression of IL-15 mitigates this fatigue, likely by enhancing mitochondrial oxidative metabolism. In vitro studies further support this mechanism, demonstrating that IL-15 improves mitochondrial function [41]. These findings suggest that inflammation underlies both central and peripheral mechanisms of fatigue in breast cancer.

#### 2.1.2 HPA axis dysfunction

The HPA axis regulates inflammatory activity through glucocorticoid synthesis and receptor sensitivity. Disruption of this axis—commonly induced by cancer and its treatment—can lead to endocrine dysfunction that promotes CRF [42]. In breast cancer, elevated estrogen levels and chronic overproduction of cortisol (CORT) can impair the hypothalamic–pituitary–ovarian (HPO) axis, resulting in abnormal regulation of estrogen and progesterone [43]. Clinical studies have documented altered CORT rhythms and concentrations in fatigued breast cancer patients, which are indicative of HPA axis dysregulation [33,[44], [45], [46], [47], [48]]. This dysregulation often coincides with reduced glucocorticoid receptor (GR) sensitivity, potentially sustaining chronic activation of pro-inflammatory pathways such as NF-κB [39]. Furthermore, inflammatory cytokines (e.g., IL-1, IL-6, TNF-α) may induce or exacerbate central fatigue through modulation of the HPA axis [49]. Taken together, these findings highlight HPA axis disruption as a central mechanism in the pathophysiology of BCRF. Importantly, because breast cancer predominantly affects females, the associated hormonal imbalances and HPA alterations may exhibit sex-specific characteristics not present in other cancers.

#### 2.1.3 ANS dysfunction

ANS dysregulation has been identified as a potential contributor to elevated inflammation in cancer survivors experiencing fatigue. As a central regulator of immune function, the ANS modulates inflammatory cytokine networks through its sympathetic and parasympathetic branches. In particular, sympathetic nervous system (SNS) activation is associated with the promotion of pro-inflammatory responses [[50], [51], [52]]. Several physiological markers—including norepinephrine, heart rate variability (HRV), salivary α-amylase activity, and catecholamine levels—have been proposed as indicators of ANS function.Clinical evidence suggests that reduced HRV is correlated with higher CRF severity in breast cancer patients, whereas improved HRV predicts more favorable fatigue outcomes [33,[52], [53], [54]]. Moreover, breast cancer survivors carrying the Met/Met genotype exhibit greater impairments in both HPA axis and SNS function, which are associated with more severe fatigue symptoms [46,55]. These findings suggest that ANS dysfunction may play an integrative role in BCRF pathogenesis by amplifying inflammatory signaling and interacting with other neuroendocrine regulatory systems.

#### 2.1.4 Circadian rhythm disruption

Disruption of the circadian rhythm is another critical contributor to BCRF. Under normal conditions, hypothalamic corticotropin-releasing factor (CRF) is secreted in response to circadian cues or psychological/physical stress, which stimulates the release of adrenocorticotropic hormone (ACTH) from the pituitary. ACTH then promotes CORT secretion from the adrenal cortex [56]. Alterations in this cascade—particularly abnormal CORT secretion—are key indicators of HPA axis dysfunction and have been associated with enhanced cytokine signaling and fatigue symptoms in various clinical contexts [47,57]. Breast cancer survivors with CRF frequently exhibit a blunted diurnal decline in CORT levels compared to non-fatigued counterparts [58]. Circadian disruption has been shown to impair immune regulation and increase the production of pro-inflammatory cytokines (e.g., IL-6, TNF-α, TGF-α), thereby exacerbating HPA axis impairment [59]. In addition, circadian dysregulation contributes directly to sleep–wake disturbances [60]. Structural brain regions involved in circadian control, such as the suprachiasmatic nucleus and thalamus, may also influence BCRF by disrupting sleep and alertness regulation [61]. Taken together, circadian rhythm disruption contributes to BCRF through a multi-system mechanism involving hormonal imbalance, immune dysregulation, and neurophysiological dysfunction.

#### 2.1.5 Gut microbiota alterations

Alterations in gut microbiota composition (dysbiosis) have been increasingly linked to the development of CRF [62]. Chemotherapy-induced dysbiosis impairs the integrity of the blood–brain barrier (BBB), leading to elevated levels of central pro-inflammatory cytokines such as IL-1β, IL-6, and TNF-α. These cytokines subsequently disrupt components of the HPA axis, including CORT and corticotropin-releasing hormone (CRH), thereby exacerbating fatigue symptoms [58,63]. Clinical studies have demonstrated that acupuncture alleviates BCRF by modulating gut microbiota, reducing the activation of inflammatory cytokines, and normalizing HPA axis hormone levels (e.g., CORT, ACTH) [64]. Supporting evidence from animal models reveals that acupuncture improves CRF by (1) increasing the abundance of beneficial bacteria such as *Candidatus Saccharimonas* and *Clostridium spp.*; and (2) decreasing the abundance of potentially pathogenic *Escherichia-Shigella* and *Streptococcus* spp [65]. These findings suggest that the gut–brain axis, particularly the microbiota–gut–HPA pathway, constitutes a key mechanism in the pathogenesis of BCRF, and that modulation of gut microbiota may represent an effective therapeutic strategy.

### 2.2 Mechanisms of peripheral fatigue

#### 2.2.1 Mitochondrial dysfunction

Cancer treatments disrupt mitochondrial structure and function through various signaling pathways, leading to impaired ATP synthesis. Given that skeletal muscle is highly metabolically active and dependent on ATP, mitochondrial dysfunction within this tissue contributes directly to peripheral fatigue [38,42,[66], [67], [68]]. Transcriptomic analyses of fatigued breast cancer patients reveal significant enrichment of mitochondrial dysfunction-related gene pathways in skeletal muscle [40]. Moreover, breast cancer cells can reprogram mitochondrial gene expression in muscle tissue independently of therapy, indicating that tumor-derived factors may initiate fatigue prior to or in the absence of treatment [69,70]. Mitochondrial dysfunction is implicated in early molecular fatigue processes, and pro-inflammatory mediators such as IL-6 have been shown to suppress mitochondrial function, leading to fatigue [71]. These findings underscore mitochondrial dysfunction as a central mechanism of breast cancer-induced skeletal muscle fatigue, driven by both tumor signals and inflammatory mediators.

#### 2.2.2 Impaired signal transmission at the neuromuscular junction (NMJ)

The neuromuscular junction, a specialized chemical synapse between motor neurons and skeletal muscle, is responsible for neuromuscular signal transmission. This process involves acetylcholine (ACh) release from presynaptic vesicles via voltage-gated Ca^2+^/K^+^ channels, binding to postsynaptic ACh receptors (AChRs), which subsequently triggers muscle contraction [72]. Studies across cancer populations, including those with breast cancer, have reported impaired NMJ transmission in patients experiencing peripheral fatigue [67]. This impairment is intrinsic to the NMJ and functionally independent of central fatigue mechanisms.

Although the precise pathophysiological mechanisms remain incompletely defined, NMJ transmission is ATP-dependent. Therefore, mitochondrial dysfunction may indirectly impair NMJ signaling by limiting ATP availability [38,42,[66], [67], [68]]. Furthermore, mitochondrial deficits are associated with NMJ denervation and fragmentation of AChRs [73]. Chronic inflammation [74] and oxidative stress [75]—both prevalent during cancer progression and treatment—may further compromise NMJ integrity. Together, these findings identify NMJ dysfunction as a key contributor to peripheral fatigue in breast cancer and a potential therapeutic target.

#### 2.2.3 Tumor-muscle energy competition

In healthy individuals, skeletal muscle utilizes glucose to produce lactate, which is either recycled locally within the muscle or converted back into glucose by the liver via the Cori cycle. In contrast, tumors in cancer patients exhibit preferential glucose uptake and convert it to lactate through aerobic glycolysis. This tumor-derived lactate is then transported to the liver, where it undergoes gluconeogenesis to regenerate glucose. The resulting glucose is preferentially recaptured by tumor cells, thereby reducing glucose availability for skeletal muscle [[76], [77], [78], [79], [80]]. This metabolic competition between tumor and muscle tissue contributes to the development of CRF. Skeletal muscle energy deficiency caused by glucose diversion impairs muscle performance and promotes fatigue. Importantly, even tumor-reducing therapies fail to restore energy balance, as they often impair mitochondrial function in metabolically active tissues such as skeletal muscle, further limiting ATP production and physical endurance [80]. Taken together, these findings suggest that tumor–muscle metabolic competition represents a novel and underexplored mechanism contributing to BCRF.

### 2.3 Summary

Persistent inflammation throughout the course of breast cancer and its treatment plays a central role in the development of both central and peripheral components of BCRF. In addition to inflammation, this review identifies two peripheral fatigue mechanisms that warrant further attention: impaired neuromuscular junction (NMJ) transmission and tumor–muscle energy competition.Beyond these primary pathways, additional contributing factors include dysregulation of the endocannabinoid system (ECS), poor dietary intake, oxidative stress [33], intramuscular lipid accumulation [40,69], and functional alterations in specific brain regions [61,81]. Preclinical evidence further suggests that enhancement of peroxisome proliferator-activated receptor gamma (PPARG) signaling may reduce lipid accumulation and improve fatigue by restoring skeletal muscle function [69]. Collectively, these findings establish BCRF as a multifactorial syndrome involving interrelated biological, metabolic, and neurological pathways. A better understanding of these mechanisms will facilitate the identification of therapeutic targets and the development of individualized interventions for BCRF.

## 3 Biomarkers of BCRF

**Fig. 2:**
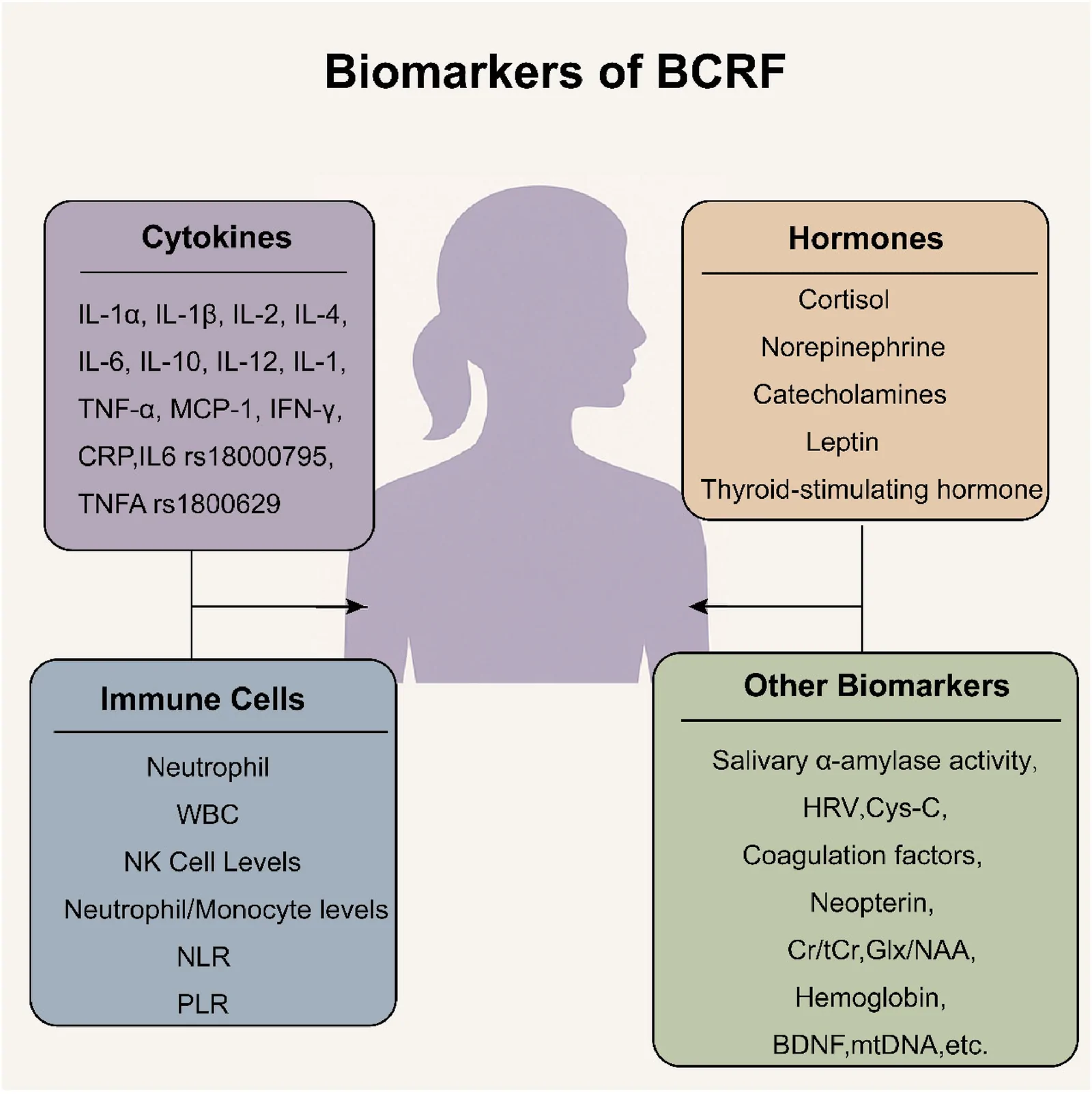
Biomarkers of Cancer-Related Fatigue in Breast Cancer. IL-1α: interleukin-1α, IL-1β: interleukin-1β, IL-1: interleukin-1, IL-2: interleukin-2, IL-4: interleukin-4, IL-6: interleukins-6, IL-10: interleukin-10, IL-12: interleukin-12, TNF-α: tumor necrosis factor-α, MCP-1: monocyte chemoattractant protein-1, IFN-γ: interferon-γ, CRP: C-reactive protein, WBC: white blood cell, NK: natural killer, NLR: neutrophil-to-lymphocyte ratio, PLR: platelet-to-lymphocyte ratio, HRV: heart rate variability, Cys-C: cystatin C, Glx/NAA: glutamate–glutamine complex to N-acetylaspartate ratio, BDNF: brain-derived neurotrophic factor.

Current research identifies biomarkers for BCRF primarily including cytokines (e.g., IL-6, TNF-α, IL-1β, IL-10), hormones (e.g., CORT, leptin, thyroid-stimulating hormone), and immune cells (such as lymphocytes, platelets, leukocytes). Additionally, plasma proteins, metabolites, ion levels, and mitochondrial DNA (mtDNA) also serve as biomarkers for BCRF (Fig. 2).

### 3.1 Cytokines

Current research has identified numerous cytokines and related biomarkers closely associated with BCRF, including IL-1α [82], interleukin-1 receptor antagonist (IL-1RA) [83], IL-1β [[83], [84], [85]], IL-2 [81], IL-4 [81,84], IL-6 [[82], [83], [84], [85], [86], [87], [88]], soluble interleukin-6 receptor (sIL-6R) [89], IL-8 [90], IL-10 [82,86], TNF-α [82,85,87,88], MCP-1 [91,92], IFNγ [82,84], and CRP [88,93]. These molecules have shown potential as biological indicators for evaluating fatigue severity in breast cancer patients. Moreover, specific genetic variants such as the IL-6 (rs1800795) and TNF (rs1800629) polymorphisms have been proposed as candidate biomarkers for fatigue susceptibility [84,94]. In addition, increased plasma levels of soluble tumor necrosis factor receptor II (sTNF-RII) within one month following chemotherapy have been positively correlated with fatigue severity among breast cancer survivors [95]. Emerging evidence also highlights MCP-1 as a promising therapeutic target, acting through the TGF-β and NF-κB pathways and functioning as a chemoattractant for macrophages and myofibroblasts [92]. Mortimer et al. [91]further reported that serum IL-12 may serve as a potential biomarker for BCRF in early-stage breast cancer. Other molecules, including vascular endothelial growth factor A (VEGF-A) and granulocyte–macrophage colony-stimulating factor (GM-CSF), have also been implicated in fatigue-related pain, suggesting a broader inflammatory context in BCRF pathogenesis [84].

### 3.2 Hormones

Hormonal dysregulation is a central feature of BCRF, largely mediated by disruptions in the HPA axis and ANS. These dysfunctions often result in altered secretion of various hormones implicated in fatigue pathophysiology.Studies have shown that plasma leptin levels increase during chemotherapy in breast cancer patients and subsequently decline after treatment. Yi Long Toh et al. [96] proposed leptin as a potential biomarker for predicting CRF severity. Notably, elevated leptin levels have been associated with increased cognitive fatigue [84]. CORT, the end product of the HPA axis, is another well-studied candidate. Lower serum CORT levels have been linked to higher CRF severity [48]. Consequently, CORT may serve as a biomarker for fatigue risk both before and after treatment [45,47]. Thyroid-stimulating hormone (TSH) [97], norepinephrine [33], and catecholamines [33] have also been proposed as potential biomarkers associated with ANS dysfunction in BCRF. These findings suggest that hormonal biomarkers reflect broader neuroendocrine imbalances and may be valuable in stratifying patients at risk for CRF.

### 3.3 Immune cells

Immune cell profiles have also been implicated in the pathophysiology of BCRF. Elevated total white blood cell (WBC) counts have been associated with increased fatigue severity in breast cancer patients [98]. Different immune cell subtypes appear to correlate with distinct fatigue dimensions. For example, emotional fatigue has been positively associated with neutrophil count, while physical fatigue demonstrates a negative association with lymphocyte count and a positive association with the platelet-to-lymphocyte ratio (PLR) [99]. In patients undergoing radiotherapy for early-stage breast cancer, higher neutrophil and monocyte counts have been linked to increased fatigue risk, whereas reduced natural killer (NK) cell levels following chemotherapy are also associated with fatigue onset [84]. Additionally, the serum neutrophil-to-lymphocyte ratio (NLR) has emerged as a promising inflammatory marker for predicting BCRF severity [48]. These findings underscore the role of systemic immune activation in the etiology of BCRF and highlight the diagnostic potential of basic hematological parameters.

### 3.4 Other biomarkers

In addition to cytokines, hormones, and immune cells, numerous other biomarkers have been associated with BCRF. Physiological markers such as salivary α-amylase activity [33], heart rate variability (HRV) [[52], [53], [54]], hemoglobin levels [[100], [101], [102]], serum albumin [103], cholinesterase activity [103], coagulation factors [100], brain-derived neurotrophic factor (BDNF) [104], serum cystatin C (Cys-C) [100], neopterin [105,106], have all demonstrated relevance to fatigue severity.

Metabolic and molecular imaging markers include total creatine (Cr + phosphocreatine, Cr/tCr) and the posterior insular glutamate–glutamine complex to N-acetylaspartate ratio (Glx/NAA), both of which are elevated in patients with CRF [107]. Electrolyte abnormalities, particularly low serum sodium levels [98], and reduced mitochondrial DNA (mtDNA) copy number [108,109]have also been implicated in fatigue pathogenesis. Increased levels of soluble intercellular adhesion molecule-1 (sICAM-1) have further been reported as markers of inflammatory activity associated with CRF [110]. In terms of genetic and epigenetic markers, polymorphisms in the BDNF gene [104] and hemoglobin-related loci [101], as well as the NFE2L2 rs2706110 TT genotype [111], have been linked to BCRF. Additionally, differential methylation at the CpG site cg22820568 within the PRDX1 gene has been proposed as an epigenetic biomarker for fatigue development [111]. Collectively, these findings support the utility of a multi-marker approach for assessing BCRF, incorporating physiological, metabolic, and genetic indicators to improve clinical prediction and patient stratification.

### 3.5 Summary

In summary, numerous biomarkers have been identified as key contributors to the pathophysiology of BCRF. These biomarkers encompass a range of physiological markers (e.g., WBC count, HRV, BDNF), metabolic indicators (e.g., creatine ratio, Glx/NAA), and genetic/epigenetic factors (e.g., BDNF polymorphisms, PRDX1 methylation). Cytokines, hormones, and immune cells also play critical roles in BCRF, with specific molecules such as IL-6, TNF-α, leptin, and CORT serving as potential diagnostic tools.The identification and validation of these biomarkers offer significant potential for improving clinical assessment and management of BCRF. Integrating multiple biomarkers—across physiological, metabolic, and genetic domains—may allow for more precise patient stratification, prediction of fatigue severity, and personalized treatment plans.Collectively, these findings underscore the complexity of BCRF and highlight the importance of continued research to identify reliable biomarkers that can guide therapeutic interventions.

## 4 Risk factors

**Fig. 3:**
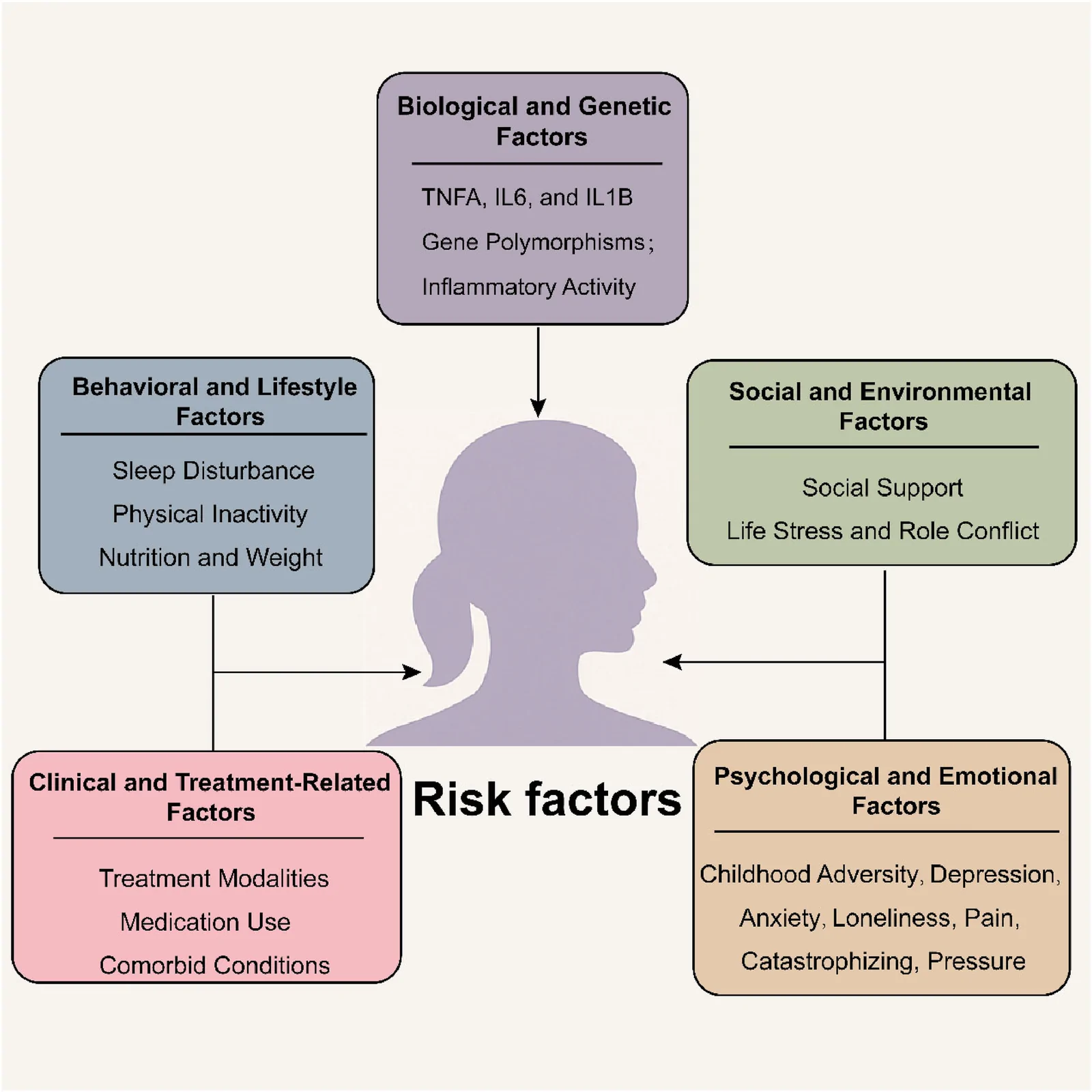
Risk factors for cancer-related fatigue in breast cancer.

BCRF is a multifactorial syndrome resulting from the complex interplay of genetic, biological, psychological, behavioral, and environmental factors. Identifying and understanding these risk factors are crucial for early screening, clinical management, and development of individualized intervention strategies(Fig. 3).

### 4.1 Biological and genetic factors

#### 4.1.1 Inflammatory gene polymorphisms

Genetic variants that regulate inflammatory signaling pathways have been implicated in BCRF susceptibility. Polymorphisms in pro-inflammatory cytokine genes such as *TNFA*, *IL6*, and *IL1B* are associated with increased fatigue in breast cancer patients undergoing radiotherapy or during survivorship [[112], [113], [114], [115]]. For instance, *TNFA-308G > A* (rs1800629) and *IL6-174G > C* (rs1800795) variants are linked to sustained fatigue post-treatment [94,116].

#### 4.1.2 Inflammatory activity

Several inflammatory biomarkers—including IL-6, CRP, IL-1RA, sTNF-RII, and TNF-α—are elevated in fatigued patients compared to non-fatigued controls. Higher levels of these cytokines are observed during and after treatment, correlating with fatigue severity [[82], [83], [84], [85], [86], [87], [88],^117^]. These findings suggest that systemic inflammation is both a marker and mediator of BCRF.

### 4.2 Psychological and emotional factors

#### 4.2.1 Childhood adversity

Childhood adversity is prevalent, reported by over 50% of adults [118]. It predicts adverse mental health outcomes in adulthood and is associated with fatigue [119,120]. A study of 270 women with early-stage breast cancer identified childhood adversity as the strongest and most consistent predictor of fatigue. Notably, 40% of these patients reported childhood physical, emotional, and/or sexual abuse or neglect [121]. Two studies of breast cancer patients undergoing active treatment further demonstrated that elevated levels of childhood adversity correlate with increased fatigue severity [122,123].

#### 4.2.2 Depression

Depression is a well-recognized and clinically important risk factor for CRF. In patients with cancer, fatigue and depression frequently co-occur; fatigue may serve as a symptom of depression, but it can also contribute to the onset of depressive states [124,125]. Research by Julienne E. Bower and colleagues has shown that a history of depression in breast cancer patients is associated with increased levels of fatigue, particularly in the emotional and cognitive domains [121,126]. In a study involving 288 early-stage breast cancer patients, a previous diagnosis of major depressive disorder significantly predicted the development of post-treatment CRF [127]. Similarly, among a cohort of 515 cancer survivors of mixed cancer types, prior depression was linked to moderate to severe fatigue, including among breast cancer survivors [20]. Moreover, a substantial body of research consistently demonstrates that depressive symptoms are positively correlated with the severity of CRF in patients with breast cancer [102,125,[128], [129], [130], [131], [132], [133], [134]]. Taken together, these findings establish depression as an independent and significant risk factor for the development and persistence of CRF in breast cancer populations.

#### 4.2.3 Anxiety

Beyond the impact of depression on BCRF, anxiety has also been identified as a significant psychological risk factor. Emerging evidence suggests that anxiety may exert an even stronger influence on CRF than depression during the early stages of cancer [135], with elevated anxiety levels consistently associated with increased fatigue prevalence [125,128,130,[132], [133], [134]]. For instance, a study involving 150 women reported significantly higher anxiety levels in breast cancer patients experiencing persistent fatigue compared to cancer-free controls [136]. Longitudinal data further demonstrate that survivors diagnosed with anxiety disorders are more likely to experience sustained fatigue over a two-year follow-up period [137]. Moreover, several studies have shown that anxiety symptoms prior to cancer diagnosis predict greater fatigue severity at 6, 12, and 24 months postoperatively [138,139]. Anxiety has also been linked to heightened morning fatigue during active cancer treatment, including both radiotherapy and chemotherapy [140]. These findings underscore the importance of assessing and managing anxiety to mitigate the risk of persistent fatigue in breast cancer populations.

#### 4.2.4 Loneliness

Loneliness, or perceived social isolation, is increasingly recognized as a significant psychosocial risk factor for BCRF [125,[131], [132], [133]]. In a cross-sectional study of BCS, Jaremka et al. [141] found a robust association between loneliness and elevated fatigue levels. Within this cohort, individuals reporting higher levels of loneliness also experienced greater pain, depressive symptoms, and fatigue compared to those with lower perceived social connectedness. Notably, lonely patients exhibited higher cytomegalovirus (CMV) antibody titers—an immune biomarker associated with heightened CRF symptomatology [141,142]. In a separate study by Kittel et al. [128] involving 118 cancer survivors, fatigue showed a particularly strong association with psychological distress among those reporting social isolation, including breast cancer survivors. These findings collectively underscore the adverse impact of loneliness on fatigue outcomes and suggest that social connectedness may play a protective role in mitigating CRF.

#### 4.2.5 Pain

Pain is a persistent and debilitating symptom that accompanies cancer progression and treatment, exerting sustained physical and psychological burdens on patients. Evidence from studies of BCS indicates a positive association between pain intensity and the severity of CRF [84]. Furthermore, research suggests that obese individuals with insulin resistance are more likely to experience frequent cancer-related pain and display heightened vulnerability to CRF-related mood and behavioral disturbances [143]. Psychological factors also play a critical role: patients experiencing psychological distress and utilizing avoidant coping strategies tend to report higher levels of both pain and fatigue [144,145]. Importantly, psychosocial interventions aimed at improving coping mechanisms have been shown to significantly reduce depressive symptoms, pain intensity, and fatigue severity—highlighting the potential of adaptive psychological states to attenuate CRF in breast cancer populations [145].

#### 4.2.6 Catastrophizing

Catastrophizing—a maladaptive cognitive process marked by diminished self-efficacy and avoidant coping in response to perceived negative outcomes—has emerged as a significant predictor of CRF across pre-treatment, treatment, and post-treatment phases. In breast cancer populations, catastrophizing has been implicated as a psychological risk factor for CRF, particularly among patients exhibiting avoidant coping strategies, who consistently report higher fatigue levels [145]. Early work by Paul B. Jacobsen and colleagues demonstrated that breast cancer patients with higher levels of fatigue catastrophizing experienced significantly greater fatigue both prior to and following radiotherapy [146]. Supporting these findings, a study involving 261 breast cancer patients at six months post-treatment identified body mass index (BMI) and catastrophizing as the two strongest predictors of persistent fatigue [147].

#### 4.2.7 Pressure

Beyond the aforementioned factors, psychological stress is recognized as an additional significant risk factor for CRF in BCS [125,[131], [132], [133],^148^]. Stress is a well-established contributor to emotional disorders such as depression and anxiety [149], which are themselves closely linked to CRF. In a structured interview-based study, Julienne E. Bower and colleagues assessed early-life stress exposure among BCS experiencing persistent fatigue and found significantly higher levels of childhood stressors in the fatigued group compared to non-fatigued controls [150]. Moreover, BCS with CRF demonstrated blunted physiological reactivity to acute psychological stress, suggesting dysregulation of stress-response systems [151]. These findings underscore the potential long-term impact of psychosocial stress on fatigue vulnerability in breast cancer populations.

### 4.3 Behavioral and lifestyle factors

#### 4.3.1 Sleep disturbance

Sleep disturbances are highly prevalent among cancer patients and are consistently associated with BCRF [82,125,[131], [132], [133]]. In breast cancer patients undergoing radiotherapy, pre-treatment sleep disruptions have been shown to predict elevated fatigue levels prior to, during, and up to six months following treatment [140]. Similarly, a longitudinal study involving 53 patients reported persistent sleep disturbances throughout chemotherapy, which coincided with a progressive worsening of fatigue symptoms. Notably, this deterioration was accompanied by increased levels of interleukin-6 (IL-6) and decreased levels of interleukin-1 receptor antagonist (IL-1RA), suggesting that inflammatory mediators may contribute to both sleep impairment and fatigue through shared pathophysiological pathways [117]. Furthermore, a comparative analysis of 232 breast cancer patients and 41 healthy controls conducted by Blickle et al. [130] revealed that poor sleep quality was significantly associated with greater fatigue severity. Collectively, these findings support the hypothesis that sleep disruption plays a central role in the development and persistence of CRF in breast cancer populations.

#### 4.3.2 Physical inactivity

Reduced physical activity has been identified as a significant predictor of elevated CRF levels in breast cancer patients [82,109]. Patients with lower activity levels tend to experience more severe fatigue post-treatment [117]. A study of long-term breast cancer survivors (LTBCS) further revealed that insufficient physical activity is associated with higher pain levels, emotional disturbances, and increased CRF severity. Notably, emotional disturbances stemming from insufficient activity also contribute to a decreased motivation for exercise, thereby exacerbating CRF [152]. Existing evidence consistently supports the beneficial effects of increased physical activity in alleviating CRF in breast cancer patients [153].

#### 4.3.3 Nutrition and weight

Findings suggest that better dietary quality and earlier eating times are associated with reduced BCRF [154], indicating that diet may play a significant role in modulating CRF in breast cancer survivors. Moreover, a study examining dietary patterns in breast cancer patients found that the onset of CRF was significantly linked to low intake of proteins, vitamins (A and E), dietary fiber, phosphorus, magnesium, potassium, iron, and copper [155]. Analysis of patients' fecal gut microbiota revealed that CRF in breast cancer may be associated with inadequate nutrient intake and poor dietary quality, potentially through gut microbiota (GM)-dependent mechanisms. In addition, several studies have highlighted the positive impact of dietary supplementation on alleviating CRF in breast cancer patients [156]. A longitudinal study of women with early-stage breast cancer identified body mass index (BMI) as one of the key predictors of fatigue severity at both 6 and 42 months post-treatment [147,157]. Collectively, these findings suggest that nutritional imbalances, including low protein intake and micronutrient deficiencies, contribute to fatigue in breast cancer patients. Furthermore, elevated BMI emerges as a consistent risk factor for CRF, potentially due to obesity-related inflammation and impaired physical function [23].

### 4.4 Social and environmental factors

#### 4.4.1 Social support

Perceived low social support is significantly associated with increased fatigue severity [130]. Lack of emotional or instrumental support contributes to psychological distress, impaired coping capacity, and biological stress activation.

#### 4.4.2 Life stress and role conflict

Stressful life events—including caregiving responsibilities, financial burden, and occupational stress—have been linked to fatigue exacerbation [117]. These external stressors interact with internal psychological states to sustain physiological stress responses and fatigue.

### 4.5 Clinical and treatment-related factors

#### 4.5.1 Treatment modalities

Different treatment types—including chemotherapy, radiotherapy, and endocrine therapy—are associated with varying fatigue profiles [117]. Higher treatment intensity and longer duration correlate with greater fatigue severity [117].

#### 4.5.2 Medication use

Certain medications, such as beta-blockers, antihistamines, sedatives, and antidepressants, may cause or worsen fatigue by affecting neurotransmitter function, cardiovascular response, or sleep architecture [117].

#### 4.5.3 Comorbid conditions

Comorbidities such as hypothyroidism, cardiovascular disease, anemia, and chronic pain syndromes are associated with increased risk and severity of BCRF [131,134,143,158]. These conditions can directly impair energy metabolism, physical functioning, or emotional stability.

### 4.6 Summary

BCRF arises from a dynamic interplay of biological, psychological, behavioral, social, and clinical factors. Key mechanisms include sustained inflammation, neuroendocrine dysregulation, emotional distress, and lifestyle impairments. Psychosocial elements such as depression, anxiety, distress, catastrophizing, childhood adversity, loneliness, and low social support play prominent roles and often interact with biological and treatment-related factors to exacerbate fatigue.Understanding and assessing these multidimensional risk profiles is essential for early detection, patient stratification, and individualized care. In the following chapter, we will review current interventions targeting these risk pathways to alleviate BCRF and improve survivorship outcomes.

## 5 Intervention strategies

**Fig. 4:**
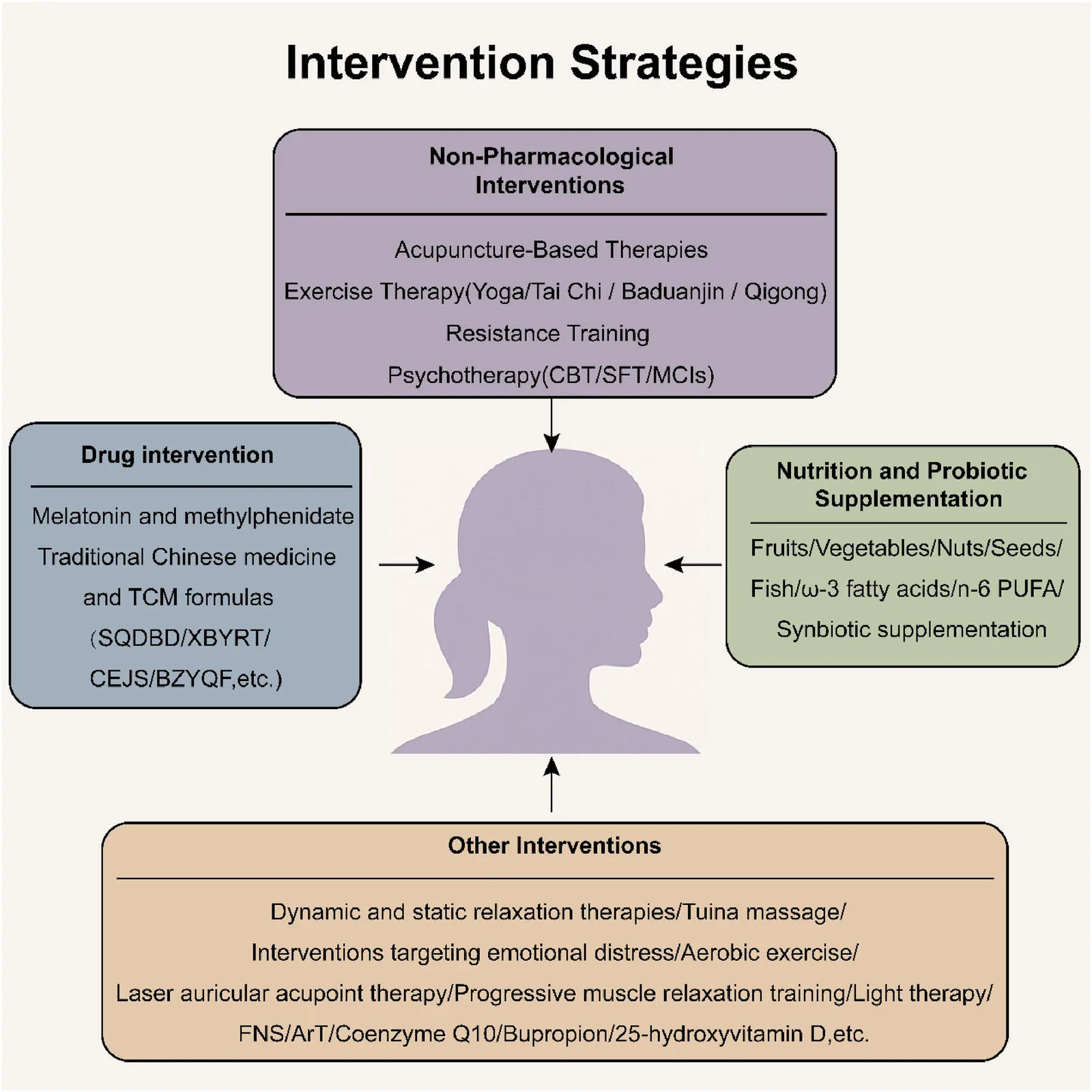
Intervention Strategies for Cancer-Related Fatigue in Breast Cancer. CBT: cognitive-behavioral therapy, SFT: solution-focused therapy, MCIs: meaning-centered interventions, TCM: Traditional Chinese medicine, SQDBD: *Scutellaria baicalensis* and Shiquan Dabu Decoction, XBYRT: Xiangbei Yangrong Decoction, CEJS: Compound Ejiao Syrup, BZYQF: Buzhong Yiqi Formula, FNS: Flexyx Neurotherapy System, ArT: Targeted Art Therapy.

Intervention strategies for BCRF encompass a broad range of non-pharmacological, pharmacological, nutritional, and integrative approaches. These interventions aim to alleviate fatigue symptoms, address underlying mechanisms such as inflammation, HPA axis dysfunction, and psychological comorbidities, and ultimately improve patients' quality of life and treatment outcomes(Fig. 4).

### 5.1 Non-pharmacological interventions

#### 5.1.1 Acupuncture-based therapies

Acupuncture has been widely utilized to alleviate chemotherapy-related symptoms, including nausea, musculoskeletal pain, anxiety, and fatigue [159]. In breast cancer patients, acupuncture has been shown to improve CRF, reduce sleep disturbances and emotional distress, relieve hot flashes, and enhance overall quality of life [[160], [161], [162], [163]]. A meta-analysis involving 18,491 cancer patients identified acupuncture as one of the most effective interventions for improving fatigue and overall well-being [164]. Xue et al. [65] demonstrated that acupuncture modulates gut microbiota and inflammatory pathways, influences CORT and ACTH secretion via the HPA axis, and reduces CRF through the microbiota–gut–brain axis. Time-point–space acupuncture (ATAS) has also shown promise in improving CRF, inflammatory markers, and sleep quality. ATAS also shows promise in improving CRF, inflammatory markers, and sleep quality [165]. Additionally, moxibustion therapy [166] and infrared laser moxibustion (ILM) [167] have also been shown to improve CRF in breast cancer patients. Importantly, much of the evidence supporting acupuncture for cancer-related fatigue comes from studies conducted in Western populations, with randomized trials demonstrating improvements in fatigue, sleep, and quality of life. This is further supported by meta-analyses including both Western and Asian cohorts. However, certain modalities, such as ATAS, have mainly been studied in East Asia. Further standardized studies are needed to confirm their applicability across different clinical settings.

#### 5.1.2 Exercise therapy

Exercise therapy, which includes practices such as yoga, qigong, tai chi, baduanjin, and resistance training, is a rehabilitative approach aimed at improving physical function through active or passive movement. Existing research has demonstrated that exercise therapy has beneficial effects on BCRF [153] and can also enhance patients' sleep quality [168]. A randomized controlled trial (RCT) found that the positive impact of exercise therapy on fatigue during adjuvant chemotherapy for breast cancer is not primarily mediated by interleukin-6 (IL-6), interleukin-1 receptor antagonist (IL-1ra), or the IL-6/IL-1ra ratio. Rather, eicosapentaenoic acid (EPA) plays a key role in improving fatigue, with higher EPA levels potentially contributing to improved muscle strength [169]. These findings suggest that exercise therapy reduces CRF in breast cancer patients and positively impacts their recovery during later stages of treatment. From a generalizability perspective, existing studies on exercise therapy for BCRF have been conducted in both Western and Asian populations, consistently supporting its beneficial effects. Conventional exercise modalities are well established in Western settings, whereas traditional mind–body practices, such as tai chi, qigong, and baduanjin, have been more extensively studied in Asia, with emerging but still limited evidence in Western populations. Further studies are needed to clarify their effectiveness and implementation across diverse populations.

##### 5.1.2.1 Yoga

Yoga, a low-to moderate-intensity mind–body exercise incorporating both physical and psychological components, has been shown to improve muscle strength and flexibility, regulate negative emotions, and enhance resilience among cancer patients [170,171]. Yoga interventions have demonstrated efficacy in reducing the expression of pro-inflammatory cytokines, enhancing immune function, and alleviating fatigue in breast cancer patients [86,172]. Moreover, yoga improves sleep quality [173], reduces psychological distress, enhances quality of life, and mitigates CRF [174]. A comparative analysis found yoga to be more effective than other exercise modalities—such as running, tai chi, and resistance training—in relieving CRF among breast cancer patients [153]. Further evidence indicates that yoga exerts particularly pronounced effects on post-treatment fatigue [175]. A large-scale study by Liu et al., involving 2423 breast cancer survivors (1252 in the yoga intervention group and 1171 in the control group), identified 220 min per week as the optimal duration for yoga to improve fatigue, quality of life, and sleep quality [176]. These findings underscore the importance of tailoring yoga-based interventions according to treatment stage, age, and other individual characteristics to maximize therapeutic outcomes.

##### 5.1.2.2 Tai chi/baduanjin/qigong

Tai chi and qigong are traditional Chinese health-preserving exercises that have gained increasing attention in oncology rehabilitation. A growing body of evidence suggests that these practices can improve fatigue, overall quality of life, emotional distress, and cognitive function in cancer patients and survivors [177,178]. They have also shown beneficial effects in alleviating BCRF [164,179]. Compared to mindfulness meditation, tai chi and qigong have demonstrated significantly greater improvements in fatigue symptoms among breast cancer patients over time [180,181]. In addition to reducing CRF, tai chi has been associated with enhanced quality of life in BCS [182]. Baduanjin, a traditional Chinese exercise with over 2000 years of history and considered a subtype of qigong, has also demonstrated therapeutic benefits. It has been shown to improve physical flexibility, reduce subcutaneous fat accumulation, enhance sleep quality, alleviate low back pain, and support mental well-being [[183], [184], [185]]. Several studies have confirmed that baduanjin interventions reduce fatigue severity and improve both sleep quality and overall quality of life in breast cancer patients [182,186,187].

#### 5.1.3 Resistance training

Resistance training, as a specific modality of exercise intervention, has been shown to exert beneficial effects in alleviating CRF [164]. A meta-analysis of nine randomized controlled trials (n = 1512) investigating the impact of resistance training on fatigue found that combined resistance and endurance training significantly reduces overall fatigue in cancer patients, including those with breast cancer. Moreover, moderate-intensity progressive programs were associated with further reductions in CRF severity [188]. In addition, resistance training has been demonstrated to improve short-term fatigue and quality of life (QoL) during anticancer treatment, while also contributing to favorable immediate QoL outcomes for patients [189].

#### 5.1.4 Psychotherapy

Psychotherapy, a therapeutic approach that utilizes psychological methods to improve psychological states or address behavioral disorders, encompasses diverse categories and forms—including cognitive-behavioral therapy (CBT), mindfulness-based therapy, and solution-focused therapy (SFT). Existing research has identified its positive role in reducing CRF [190,191].

##### 5.1.4.1 CBT

CBT is a patient-centered psychological intervention that actively involves both patients and caregivers in the therapeutic process. It has been identified as one of the most effective strategies for alleviating CRF in breast cancer patients [168,192]. A specific subtype, cognitive-behavioral therapy for insomnia (CBT-I), is widely recommended by leading authorities including the American Academy of Sleep Medicine (AASM), the American College of Physicians (ACP), and the European Sleep Research Society (ESRS) as an evidence-based treatment for cancer-related insomnia [[193], [194], [195], [196]]. Two studies by Krista M. Greeley and colleagues demonstrated that CBT-I significantly reduces CRF in breast cancer patients with comorbid insomnia, potentially through mediating effects on sleep quality. These findings suggest that improvements in insomnia symptoms may serve as a pathway through which CBT-I alleviates fatigue [197,198].

##### 5.1.4.2 SFT

SFT, a psychoeducational and supportive intervention, has gained increasing recognition for its potential in mitigating cancer-related complications. An integrative study by Maryam Maleki et al. [199] demonstrated that SFT is effective in reducing stress, anxiety, depression, somatization, CRF, pain catastrophizing, and maladaptive coping behaviors. In parallel, it was shown to enhance psychological well-being, hope, adaptive coping strategies, self-efficacy, overall quality of life, and sexual quality of life in cancer patients. A recent meta-analysis further confirmed the positive impact of SFT on CRF specifically in breast cancer patients, with no significant adverse effects reported, suggesting that SFT may serve as a safe and effective adjuvant intervention for managing CRF in this population [200].

##### 5.1.4.3 Meaning-centered interventions (MCIs)

MCIs, a psychotherapeutic model integrating existential psychology and positive psychology, aim to help individuals cope with adversities and enhance mental health by reconstructing their cognitive perception of life meaning. Existing research has shown that MCIs can improve quality of life, alleviate existential distress, reduce depressive symptoms, and mitigate CRF in breast cancer patients [85,201].

### 5.2 Drug intervention

#### 5.2.1 Melatonin and methylphenidate

Melatonin, a low-cost and widely accessible natural supplement, has demonstrated radioprotective properties in animal models and is considered safe for human use. It is known to reduce pro-inflammatory immune responses, regulate circadian rhythms and sleep quality, and protect against mitochondrial oxidative stress [202,203]. Several studies have reported that oral melatonin significantly improves CRF in breast cancer patients [204]; however, appropriate dosing should account for cancer stage and treatment modality [205]. Notably, a recent double-blind, placebo-controlled phase III trial found that melatonin did not prevent or significantly reduce fatigue or other symptoms in patients with early-stage breast cancer undergoing radiotherapy [206]. These findings suggest that melatonin does not exert uniform benefits across all cancer types and clinical contexts, highlighting the importance of individualized treatment decisions based on cancer type, stage, and therapy regimen [207].Methylphenidate, a central nervous system stimulant and one of the most studied pharmacologic agents for CRF, has shown efficacy in improving fatigue in breast cancer patients [207]. However, results have been inconsistent across other cancer populations, with some studies reporting limited or no effect on fatigue severity [[208], [209], [210], [211]].

#### 5.2.2 Traditional Chinese medicine (TCM) and its formulas

TCM and TCM formulas have demonstrated promising efficacy in alleviating CRF without significant adverse effects. Numerous studies have reported that *Panax ginseng* [212,213] and *Panax quinquefolius* [214] significantly reduce CRF in breast cancer patients, with their effects surpassing those of methylphenidate [207]. *Scutellaria baicalensis* and Shiquan Dabu Decoction (SQDBD) also show efficacy in alleviating postoperative fatigue symptoms in breast cancer patients [212]. Additionally, modified Jiawei Xiangbei Yangrong Decoction (XBYRT) [214], Astragalus polysaccharide (PG2) [215], Compound Ejiao Syrup (CEJS) [216,217], and Buzhong Yiqi Formula (BZYQF)—which includes *Astragalus membranaceus*, *Panax ginseng*, *Atractylodes macrocephala*, *Glycyrrhiza uralensis*, *Citrus reticulata*, *Angelica sinensis*, Cimicifuga foetida, and *Bupleurum chinense*—demonstrate notable safety and efficacy in managing CRF in breast cancer patients [218]. Furthermore, *Ganoderma lucidum* spore powder (GLSP) has been shown to improve both CRF and quality of life (QoL) in breast cancer patients undergoing endocrine therapy, with no significant adverse effects reported [219]. In other oncology studies, Shenqi Fuzheng Injection (SFI) has demonstrated significant improvement in cancer-related muscle fatigue (CRMF), potentially through mechanisms involving AMP-activated protein kinase (AMPK) activation and phosphatidylinositol 3-kinase/protein kinase B (PI3K/Akt) inhibition, which enhance mitochondrial function in myocytes and modulate the expression of mitochondrial metabolic enzyme manganese superoxide dismutase (MnSOD) and apoptosis-related proteins Bcl-2-associated X (Bax) and B-cell lymphoma 2 (Bcl-2) [220]. These findings underscore the therapeutic potential of TCM and TCM formulas in managing CRF in breast cancer patients.

### 5.3 Nutrition and probiotic supplementation

Nutritional interventions, including diets rich in fruits, vegetables, nuts, seeds, and fish, have been proposed as potential strategies for improving BCRF [221,222]. A nationwide study by Amber S. Kleckner et al. [223] found that better nutritional status was associated with greater increases in circulating ω-3 fatty acids following fish oil supplementation, and these increases in circulating ω-3 fatty acids were linked to more significant improvements in fatigue. Additionally, another study demonstrated that guarana extract and diets enriched with whole foods, ω-3 fatty acids, fruits, and vegetables can effectively manage CRF in breast cancer patients [156]. Research by Luke J. Peppone et al. [224] revealed that n-6 polyunsaturated fatty acid (n-6 PUFA) supplementation significantly reduced pro-inflammatory markers in the TNF-α signaling pathway, thereby alleviating CRF in breast cancer patients. A systematic review by Silvia Belloni et al. further indicated that probiotic supplementation improved fatigue in both chemotherapy-treated and post-chemotherapy colorectal cancer patients. Moreover, synbiotic supplementation was shown to significantly reduce fatigue in breast cancer patients undergoing chemotherapy [225].

### 5.4 Other interventions

Beyond the aforementioned approaches, various complementary and integrative interventions have demonstrated beneficial effects in alleviating BCRF. These include both dynamic and static relaxation therapies [226], tuina massage [179], interventions targeting emotional distress [227], aerobic exercise [179], laser auricular acupoint therapy [228], progressive muscle relaxation training [229], light therapy [230], and music therapy combined with palliative care [231]. In addition, emerging modalities such as cardiac magnetic stimulation, optical stimulation [232], Flexyx Neurotherapy System (FNS) [233], and Targeted Art Therapy (ArT) [234] have shown preliminary efficacy in reducing fatigue symptoms. Several nutritional and pharmacologic agents—such as coenzyme Q10 [235], bupropion [236], 25-hydroxyvitamin D [237], and guarana [238]—have also been associated with improvements in BCRF. Among pharmacological candidates, bupropion, a norepinephrine–dopamine reuptake inhibitor (NDRI), has progressed from pilot evidence, initially established in a randomized placebo-controlled trial by Jim et al. [236], to a nationwide phase III confirmatory study. Recently, Peppone et al. reported primary outcomes demonstrating that bupropion significantly alleviates cancer-related fatigue in survivors [239], which represents the first positive large-scale pharmacological trial in this field in over a decade. Proposed underlying mechanisms include NDRI-mediated dopaminergic modulation, restoration of hypothalamic-pituitary-adrenal (HPA) axis function, and anti-inflammatory effects [240]. Notably, multimodal combination therapies, which integrate multiple interventions, appear to be more effective than single approaches in managing CRF among breast cancer patients [164].

### 5.5 Summary

Interventions for BCRF span behavioral, psychological, pharmacological, and nutritional domains. Acupuncture, exercise, and psychotherapy stand out as the most evidence-supported non-pharmacologic approaches. Pharmacologic treatments, including melatonin, methylphenidate, and TCM, show potential but require further validation in controlled studies. Nutrition and microbiota-targeted strategies offer new directions for systemic fatigue regulation. Future clinical applications should prioritize personalized, multimodal programs based on individual fatigue mechanisms and comorbidities.

## 6 Conclusion

This review comprehensively synthesized the current evidence regarding the pathogenesis, biomarkers, risk factors, and intervention strategies of BCRF, aiming to inform both clinical practice and future research.Mechanistically, BCRF is a multifactorial syndrome involving both central and peripheral fatigue pathways. Central mechanisms encompass inflammatory signaling, dysregulation of the HPA axis, ANS dysfunction, circadian rhythm disruption, gut microbiota imbalance, and alterations in brain function. Peripheral mechanisms primarily include mitochondrial dysfunction, neuromuscular transmission deficits, and tumor-muscle metabolic competition. Additional contributing factors include oxidative stress, lipid accumulation, and endocannabinoid system (ECS) dysregulation.

Importantly, BCRF is shaped by complex interactions among biological, psychological, behavioral, and hormonal dimensions. As breast cancer predominantly affects women, estrogen signaling plays a sex-specific modulatory role in fatigue pathogenesis, particularly via HPA axis regulation and inflammatory pathways.

Numerous biomarkers—including cytokines (e.g., IL-6, TNF-α), CORT, leptin, immune cell ratios, and mitochondrial DNA—have been identified to correlate with BCRF severity, offering potential targets for assessment and individualized risk stratification. These biomarkers exhibit dynamic changes across disease stages and treatment modalities, emphasizing the need for context-specific clinical evaluation.

Psychological and behavioral risk factors—such as depression, anxiety, childhood adversity, loneliness, catastrophizing, physical inactivity, and sleep disruption—are critical determinants of fatigue onset and persistence. They interact with immune, endocrine, and neurophysiological systems, reinforcing the need for integrative biopsychosocial models in BCRF research and care.

Multiple intervention strategies have demonstrated efficacy in alleviating BCRF. Non-pharmacological approaches—such as acupuncture, exercise therapy (e.g., yoga, tai chi), and psychological interventions (e.g., cognitive-behavioral therapy, solution-focused therapy, meaning-centered therapy)—exhibit robust and durable effects across physical and emotional fatigue domains. Pharmacological interventions, including melatonin, methylphenidate, and traditional Chinese medicine (TCM), show promise but require further validation regarding optimal indications, dosage, and mechanisms of action. Nutritional interventions, particularly omega-3 fatty acid supplementation and microbiota-targeted therapies (e.g., probiotics, synbiotics), are emerging as safe and systemically beneficial strategies.

In conclusion, BCRF represents a complex, multifactorial condition necessitating interdisciplinary and personalized management. Future research should focus on: 1)Elucidating mechanism-specific fatigue phenotypes (e.g., inflammation-driven vs. endocrine-driven); 2)Developing clinically validated biomarker panels for early detection and treatment monitoring; 3)Designing integrative, multimodal intervention programs tailored to individual risk profiles and symptom trajectories.Such efforts are essential for reducing symptom burden, enhancing quality of life, and optimizing survivorship outcomes for breast cancer patients and survivors.

## CRediT authorship contribution statement

**Chaofan Zhao:** Writing – original draft, Methodology, Formal analysis, Conceptualization. **Qi Qiu:** Writing – original draft, Visualization, Investigation, Data curation. **Yuanyuan Wang:** Resources, Investigation, Data curation. **Yidi Jiang:** Validation, Software, Formal analysis. **Kunshuang Shen:** Project administration, Investigation. **Le Yang:** Supervision, Methodology. **Ye Sun:** Validation, Software. **Zhengcan Zhou:** Resources, Investigation. **Mingqian Jia:** Writing – review & editing, Data curation. **Haibin Liu:** Writing – review & editing, Supervision, Funding acquisition. **Hui Sun:** Writing – review & editing, Supervision, Methodology. **Ning Zhang:** Supervision, Project administration, Funding acquisition, Conceptualization.

## Funding

National Multidisciplinary Innovation Team Project in Traditional Chinese Medicine(ZYYCXTD-D-202407); Investigating the Pharmacodynamic Components and Quality Markers of Fufang E'jiao Jiang in Alleviating Cancer-Related Fatigue and Syndrome Based on Chinmedomics(22042240002); Heilongjiang Provincial Key Research and Development Plan (2022ZX02C04).

## Declaration of competing interests

None Declared.
